# A new species of *Monstrillopsis* (Crustacea, Copepoda, Monstrilloida) from the lower Northwest Passage of the Canadian Arctic

**DOI:** 10.3897/zookeys.709.20181

**Published:** 2017-10-18

**Authors:** Aurélie Delaforge, Eduardo Suárez-Morales, Wojciech Walkusz, Karley Campbell, C. J. Mundy

**Affiliations:** 1 Centre for Earth Observation Science (CEOS), Faculty of Environment, Earth and Resources, University of Manitoba, Winnipeg, Manitoba, Canada R3T 2N2; 2 El Colegio de la Frontera Sur (ECOSUR), Unidad Chetumal. P.O. Box 424. Chetumal, Quintana Roo 77014. Mexico; 3 Department of Fisheries and Oceans, Winnipeg, Manitoba, Canada R3T 2N6

**Keywords:** copepods, zooplankton, taxonomy, under-ice community, Canadian Arctic Archipelago

## Abstract

A new species of monstrilloid copepod, *Monstrillopsis planifrons* **sp. n.**, is described from an adult female that was collected beneath snow-covered sea ice during the 2014 Ice Covered Ecosystem – CAMbridge bay Process Study (ICE-CAMPS) in Dease Strait of the Canadian Arctic Archipelago. Currently, up to six species of this order are known to occur in polar latitudes. The new species described herein shares similarities with *Monstrillopsis dubia* (Scott, 1904) but differs in its body proportions and cephalothorax ornamentation; the cephalothorax is covered by minute scattered papillae on dorsal and ventral surfaces; this species has a reduced fifth leg endopod, fifth leg exopod armed with three setae, antennule with fused segments 3–4, and the genital double-somite bears unique posterolateral processes. This is the second species of this genus recorded in the Arctic, after *Monstrillopsis ferrarii* (Suárez-Morales & Ivanenko, 2004), described from the White Sea, and is the first record of *Monstrillopsis* in Canadian waters. With the addition of this new species and the recognition of *Monstrillopsis bernardensis* comb. nov. as a member of this genus, the number of nominal species is now 15. Overall, this genus has a tendency to be distributed in temperate and cold waters, while only three species have been found in tropical and subtropical latitudes.

## Introduction

Members of the marine copepod order Monstrilloida (Sars, 1901) are rarely obtained during plankton surveys as the first naupliar developmental stage and the non-feeding adults are free-living forms that are only briefly present in the water column and can be caught during plankton samplings in coastal areas (Suárez-Morales and Dias 2001; Suárez-Morales 2001). Their postnaupliar and juvenile stages are endoparasites of different groups of benthic invertebrates, including polychaetes, molluscs, and sponges (Huys et al. 2007; Suárez-Morales et al. 2010, 2014). In the Arctic, the Monstrilloida have been recorded occasionally, but because of their low abundances in the plankton and lack of expertise in their identification, assessments have been largely reduced to reporting the presence of these copepods in some samples (Walkusz et al. 2009, 2010).

The biology and diversity of the planktonic fauna living beneath sea ice are still being studied in the Arctic regions. There have been surveys on the dynamics and composition of planktonic copepods (Kosobokova and Hopcroft 2010; Walkusz et al. 2010, 2013; Weydmann et al. 2013), but data on the Monstrilloida remains limited. The order Monstrilloida is currently represented by five genera containing more than 160 species. However, there are extended geographic areas in which the presence of monstrilloid copepod fauna remains unknown (Suárez-Morales 2011). As a result, local and regional lists are expected to grow as the exploration of under-sampled regions continues (Suárez-Morales 2011, Suárez-Morales and McKinnon 2014, 2016; Lee et al. 2016). Currently, there are only a few species of monstrilloids known from Arctic or Subarctic waters (Fontaine 1955; Davis and Green 1974; Suárez-Morales and Ivanenko 2004). These species include two of *Monstrilla* (i.e., *M. nasuta* Davis & Green, 1974 and *M. arctica* Davis & Green, 1974), and three of *Monstrillopsis* (*M. dubia* Scott, 1904, *M. ferrarii* Suárez-Morales & Ivanenko, 2004, and *M. bernardensis* (Willey, 1920), comb. nov.). The latter species was originally described as *Thaumaleus bernardensis* but it is clearly a species of *Monstrillopsis* by its possession of four caudal setae and a modified male fifth antennulary segment (see Willey 1920, figs 68–70).

As part of the 2014 Ice Covered Ecosystem – CAMbridge bay Process Study (ICE-CAMPS) in Dease Strait, Canadian Arctic, zooplankton samples were collected between March and June. Amongst these samples, adult female individuals of the monstrilloid copepod genus were collected. Taxonomic examination of one of these specimen revealed that it represented a previously undescribed species of *Monstrillopsis* (sensu Sars, 1921). The purpose of this study is to describe this species, to compare it with its closest congeners, and provide insight on the diversity and distribution of the genus *Monstrillopsis* in Dease Strait, NU, Canada.

## Materials and methods

**Figure 1.**
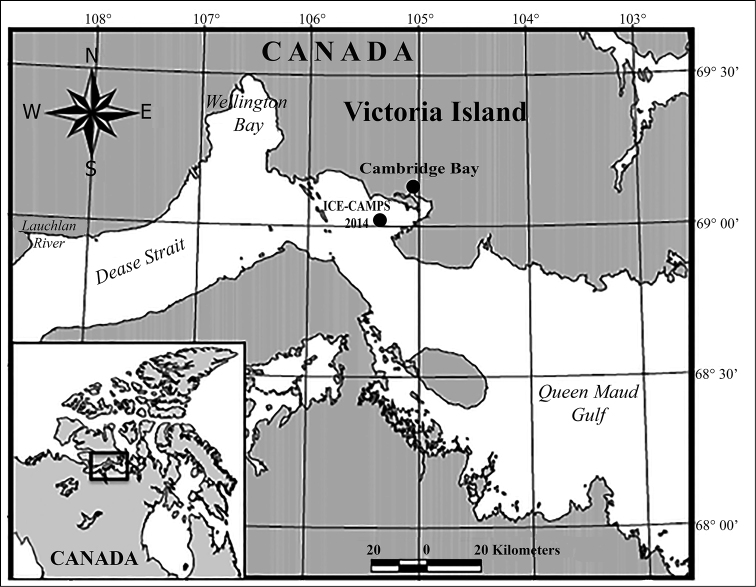
Map of study site location in Dease Strait – ICE-CAMPS 2014 (Ice Covered Ecosystem – CAMbridge bay Process Study), near Cambridge Bay, lower Northwest Passage, Canadian Arctic.

The specimen observed here was obtained during the 2014 ICE-CAMPS campaign in Dease Strait, lower Northwest Passage of the Canadian Arctic (Fig. 1). The sample obtained on 02 June 2014 contained an unidentified female monstrilloid copepod of the genus *Monstrillopsis*. Zooplankton were collected by performing vertical hauls with a standard WP2 plankton net (100 µm mesh size, 50 cm mouth diameter). Samples were fixed and preserved in 4% buffered formalin. The specimen was sorted out and processed for identification. The separation and preliminary observations were made under an Olympus SZX 16 stereomicroscope and a Leica CME compound microscope. The specimen was placed in glycerol and lightly stained with Methylene Blue before partial dissection. The dissected appendages, mainly the legs 1–4, and the remaining parts of the body (i.e., cephalothorax and urosome) were mounted on slides using glycerol as mounting medium and sealed with acrylic nail varnish. Drawings were prepared at 200–1000× magnifications with the aid of a camera Lucida mounted on an Olympus BX51 compound microscope equipped with Nomarski DIC. The description, including the terminology of the antennular armature follows the descriptive standards set by Grygier and Ohtsuka (1995).

## Results

### Order Monstrilloida Sars, 1903

#### Family Monstrillidae Dana, 1849

##### Genus *Monstrillopsis* Sars, 1921

###### Monstrillopsis planifrons sp. n.

Taxon classificationAnimaliaMonstrilloidaMonstrillidae

http://zoobank.org/F89104C6-A157-4598-8536-D2A2FE976B3F

Figs 2 , 3 , 4

####### Material examined.

Adult female holotype from Dease Strait (69°1.5'N; 105°20.2'W), partially dissected. Selected appendages (legs 1–4) and cephalothorax and urosome on semi-permanent slides, mounted on glycerine. Date of collection: 02 June 2014. Plankton sampled underneath ice-covered water column. Slides deposited in the Collection of Zooplankton at El Colegio de la Frontera Sur (ECOSUR), in Chetumal, Mexico (ECO-CH-Z-09535).

####### Description.

**Figure 2.**
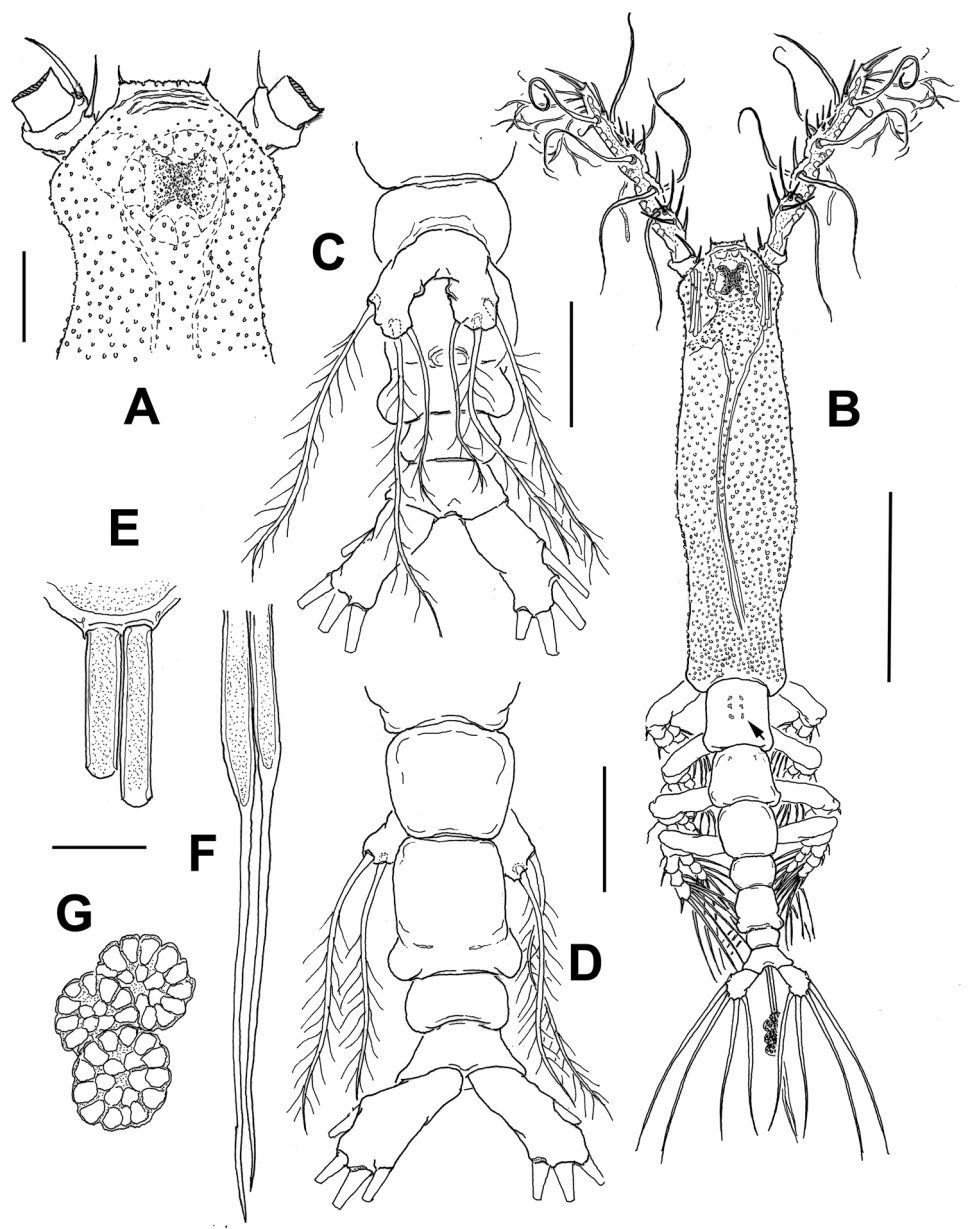
*Monstrillopsis planifrons* sp. n., adult female holotype from the Canadian Arctic. **A** cephalic region, dorsal view **B** habitus, dorsal view, arrow shows three paired dorso-medial papilla-like processes on second pedigerous somite **C** urosome, ventral view, showing fifth legs, ovigerous spines not shown, only its insertion area **D** urosome, dorsal view **E** insertion of ovigerous spine on ventral surface of genital double-somite **F** terminal section of ovigerous spines **G** eggs along ovigerous spines. Scale bars: **A, C, D** 100 µm, **B** 500 µm, **E–G** 25 µm.

**Figure 3.**
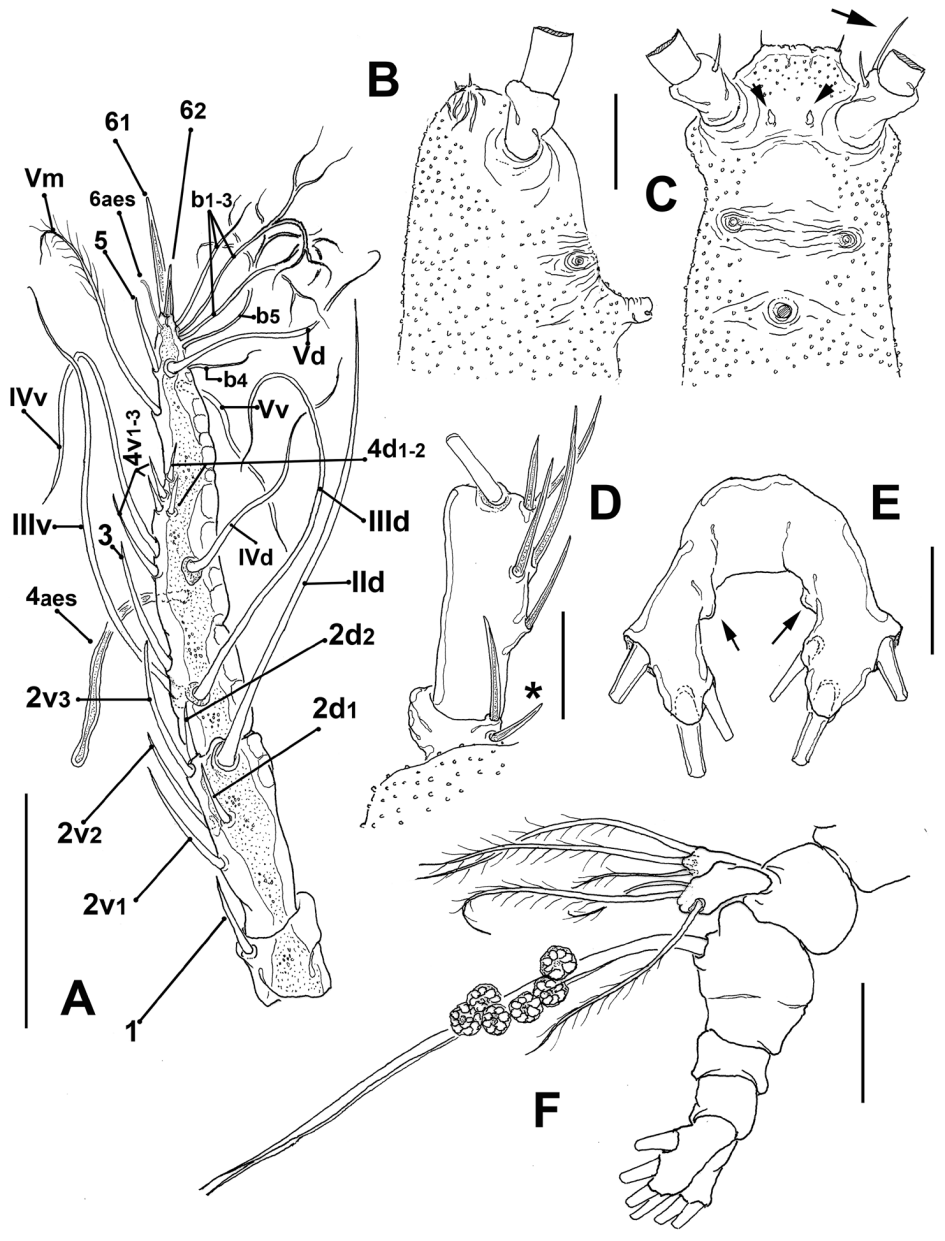
*Monstrillopsis planifrons* sp. n., adult female holotype from the Canadian Arctic. **A** right antennule showing armature following nomenclature by Grygier & Ohtsuka (1995), dorsal view **B** cephalic area showing forehead and perioral ornamentation, lateral view **C** cephalic area, showing cuticular processes and ornamentation, ventral view, arrow shows supernumerary spiniform element on first segment of left antennule **D** detail of first and second antennulary segments of left antennule showing supernumerary spiniform element on first segment (*) **E** fifth leg, ventral view showing small lobe-like processes on inner margin of exopodal (outer) lobes (arrowed) **F** urosome, showing fifth legs and ovigerous spines, lateral view. Scale bars: **A** 200 µm, **B–F** 100 µm, **D–E** 50 µm.

*Female*. Body length of holotype specimen measured from anterior end of cephalosome to posterior margin of anal somite = 1.92 mm. Cephalothorax (incorporating first pedigerous somite) approximately 1.1 mm long, representing 58% of total body length (Fig. 2B). Oral papilla located at 20% of way back along ventral surface of cephalothorax. Pair of relatively large ocelli present, pigment cups moderately developed, medially conjoined, strongly pigmented; ventral cup larger than lateral cups (Fig. 2A). Cephalic area with conspicuous, protruding “forehead” process with flat, coarse anterior margin ornamented with transversely arranged cuticular ridges at its base (Figs 2A, 3C). Protruding frontal process with pair of sensilla inserted at each side. Cephalic ventral ornamentation including: 1) a pair of small papilla-like structures between antennule bases (paired arrows in Fig. 3C), 2) preoral pair of nipple-like processes on anterior ventral surface; processes with adjacent wrinkles, rounded in shape; processes connected medially by transverse wrinkles (Fig. 3C). Cephalothorax covered with small papilla-like cuticular processes arranged randomly on ventral, lateral and dorsal surfaces (Figs 2B, 3B, C). Other ventral cuticular ornamentation including shallow striae on lateral and anterior surfaces of oral cone. Pedigerous somites 2–4 measuring 0.47 mm representing 24.7% of total body length; second pediger ornamented dorsally with three pairs of minute papilla-like processes on medial position (arrowed in Fig. 2B). Third pediger with pair of small sensilla on anterior half (Fig. 2B).

Urosome slender, consisting of fifth pedigerous somite, genital double-somite, and preanal and anal somites, together measuring 0.31 mm and representing 16% of total body length. Relative lengths of fifth pedigerous somite, genital double-somite, preanal and anal urosomites as: 31:38.2:14.1:16.7= 100, respectively (Fig. 2C, D). Genital somite longest of urosome, with pair of rounded expansions on posterolateral margins; surface smooth (Fig. 2C, D); somite with rounded ventral protuberance on anterior margin and with shallow suture on lateral surface (Fig. 3F). Ovigerous spines arising ventrally from proximal half of genital double-somite; spines paired, relatively short (0.62 mm), about 0.24% of total body length, posteriorly reaching distal margin of caudal setae (Fig. 2B). Spines basally separate, slender, straight at base and along shaft, both distally tapering into acute points; left spine slightly shorter (Fig. 2E, F). Specimen with a few eggs attached to ovigerous spines (Fig. 2G). Caudal ramus subrectangular, 1.9 times longer than wide, armed with four subequally long lightly setulated caudal setae (Fig. 2B).

Antennules relatively long, slender, not straight but clearly divergent (Fig. 2B). Antennule length = 0.59 mm, representing about 31% of total body length and 51 % of cephalothorax length; antennule indistinctly 4-segmented, segments 3–4 partially fused. Relative length of segments, from base to top as: 12.1; 29.2; 17.1; 41.6. Last segment distally tapering (Fig. 3A). Antennulary armature in terms of pattern described by Grygier and Ohtsuka (1995), including setae (Roman numerals), spines (Arabic numerals), and aesthetascs as: element 1 present on first segment, represented by relatively long, spiniform; elements on second segment: 2d_1-2_, 2v_1-3_ and long seta IId reaching slightly beyond distal end of antennule. Third segment with elements 3, IIId, and IIIv; element 3 remarkably long. Segment four bearing elements 4d_1,2_, 4v_1-3_, element 4v_1_ longest of group. Setae IVd, IVv, Vd, Vv, Vm, and 4aes present. Element 5 spiniform. Subterminal elements b_1-5_, branched; 6aes present. Apical elements 6_1_ and 6_2_ strong, spiniform; 6_1_ twice as long as 6_2_ (Fig. 3A). First segment of left antennule with an additional spine about half as long as element 1; supernumerary spine absent on right antennule.

**Figure 4.**
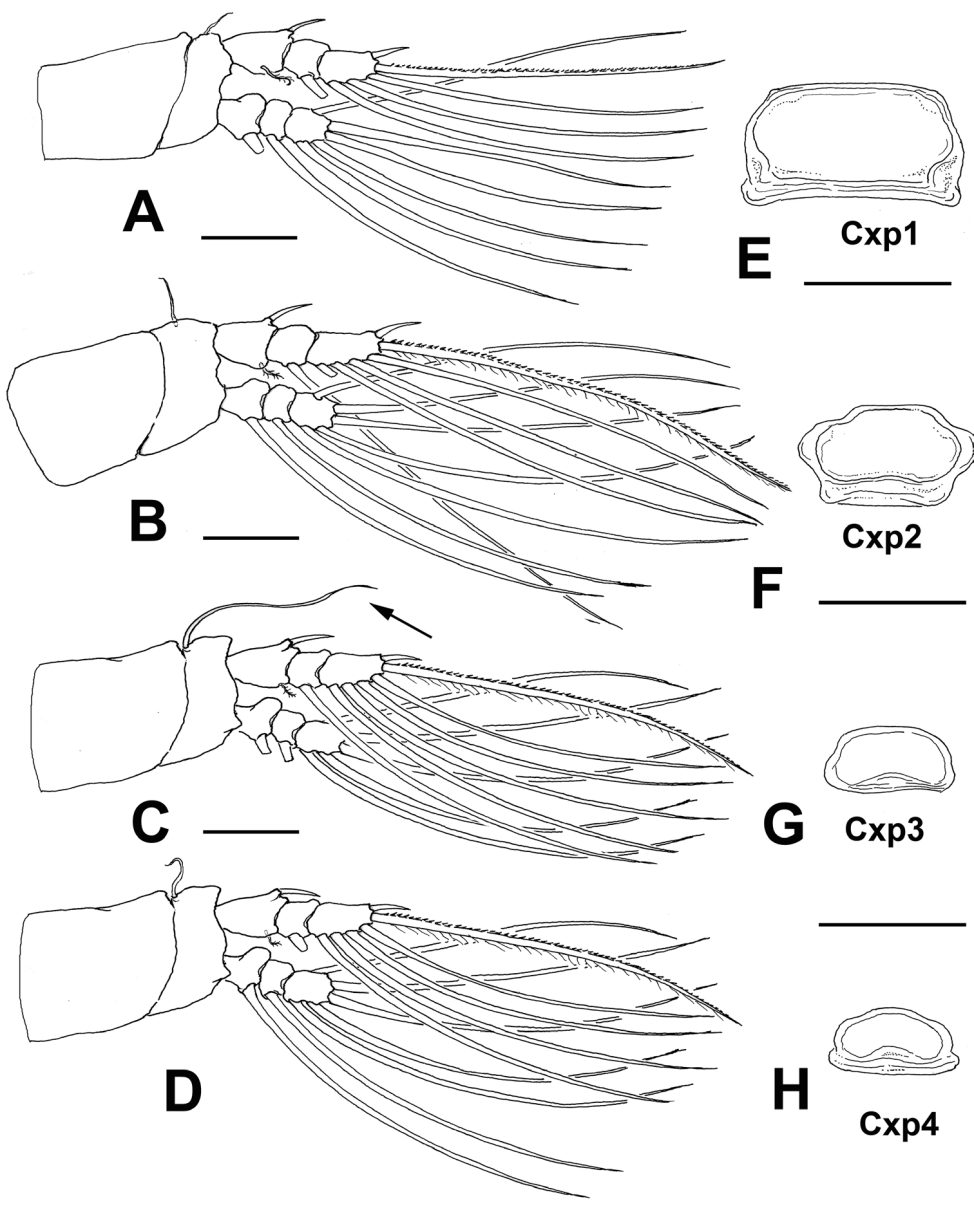
*Monstrillopsis planifrons* sp. n., adult female holotype from the Canadian Arctic. **A** first leg **B** second leg **C** third leg with longer basipodal seta **D** fourth leg **E–H**. intercoxal sclerites of legs 1–4, respectively. Scale bars: **A–D** 100 µm, **E–H** 50 µm.

Incorporated first pedigerous somite and succeeding three free pedigerous somites each bearing a pair of biramous legs. Legs 1–4 slightly increasing in size posteriorly, leg 1 being shortest. Intercoxal sclerites of legs 1–4 subrectangular, widest transversely, with rounded margins, with surface and posterior margins smooth; sclerites with decreasing size, that of leg 1 being largest (Fig. 4AE–H). Basis of legs articulating with large, rectangular coxa along diagonal line. Basis of legs 1–4 with hair-like lateral seta (Fig. 4A–D); on leg 3, this seta about 6 times longer and slightly thicker than those on the other legs (arrowed in Fig. 4C). Endopodites and exopodites of legs 1-4 triarticulated. Ramus setae all biserially plumose except spiniform outer seta on exopodal segments 1 and 3, and inner seta of first exopodal segment, these latter being short and sparsely setulated (Fig. 4A–D). Spine on exopodal segment 1 of legs 1–4 long, almost as long as segment, reaching distal margin of succeeding segment. Long apical exopodal setae of legs 1–4 with inner margin setulated, inner margin lightly spinulose.

Armature formula of legs 1-4 as:

**Table:** 

	basis	endopod	exopod
leg 1	1-0	0-1;0-1;1,2,2	I-1;0-1;I,2,2
legs 2–4	1-0	0-1;0-1;1,2,2	I-1;0-1;I,1,2,2

Fifth legs medially conjoined, indistinctly bilobed, inner (endopodal) lobe incospicuous, represented by small inner protuberance (arrows in Fig. 3E). Outer lobe large, robust, with rounded margins; lobe armed with three subdistal setae. Innermost seta shortest, all fifth leg setae biserially and lightly setulated (Fig. 2C).

####### Etymology.

The specific epithet, derived from the Latin adjective *planus* (flat) and the noun *frons* (forehead), makes reference to the flat, protuberant frontal margin on the cephalic area, which is distinctive of this species.

####### Remarks.

The Arctic species described herein is assigned to the genus *Monstrillopsis* owing to its possession of the combination of characters noted by Sars (1921) in his diagnosis of this genus: 1) two free somites posterior to the genital double-somite, 2) eye fully developed, 3) four-segmented antennules in the female, 4) oral papilla occurring near the anteriormost part of the cephalothorax: < 20% of way back along cephalothorax, 5) bilobed female fifth leg, outer lobe armed with three setae, 6) furcal rami with four setae. For the *Monstrillopsis* genus, the number of female caudal setae is four, as in the present new species. However, males of some species can have more caudal setae (i.e., *Monstrillopsis reticulata* (Davis, 1949), *M. zernowi* Dolgopolskaya, 1948) (Suárez-Morales et al. 2006), but except for the aberrant *M. zernowi*, with 5 caudal setae, the character is consistently present in the females.

*Monstrillopsis planifrons* sp. n. differs in several respects from its known congeners. Most species of *Monstrillopsis* of which females are known, like *M. dubioides* Suárez-Morales, 2004, *M. ferrarii* Suárez-Morales & Ivanenko, 2004, and *M. chilensis*, have affinities with *M. dubia*. This group of species share a relatively short cephalothorax (ca. 50–56% of total body length), four caudal setae, a well-defined digitiform inner lobe on the female fifth leg, an outer lobe armed with three long setae, antennule relatively short (30–35% of cephalothorax length) distinctly 4-segmented, and a relatively long genital double-somite (ca. 30% of urosome) with an expanded proximal half (Suárez-Morales et al. 2006, 2008).

*Monstrillopsis planifrons* sp. n. differs from this group of species in having relatively longer antennules (53% of total body length), which is one of the most striking characters of this species; this pattern is similar to that depicted by Dolgopolskaya (1948, fig. 3.1) for *M. zernowi*. This aberrant species also resembles the new species in having fused antennulary segments 3–4. The new species clearly diverges from *M. zernowi* in the number of caudal setae: 5 vs. 4 in the new species. In addition, the fifth leg has a well-defined inner lobe (Dolgopolskaya 1948, fig. 3.5), thus diverging from the weakly developed inner process found in the new species. Also, in *M. zernowi* the frontal anterior margin of the cephalic region is depressed, with a medial protuberance (Dolgopolskaya 1948, fig. 3.2), thus differing from the produced, flat process that is present in *M. planifrons* sp. n. A relatively long antennule (47% of cephalothorax length) is also present in *M. dubia* (Scott 1904, pl. XIII, fig. 14).

The antennule structure and armature are also a source of distinctive apomorphies of this new species. In several species of the genus the female antennulary segments 3–4 are clearly separate, as in *M. dubia* (Scott 1904, pl. XIII, fig. 14), *M. ferrarii* (Suárez-Morales and Ivanenko 2004, fig. 4B), *M. dubioides* (Sars 1921; Suárez-Morales and Ivanenko 2004, fig. 9A), *M. chilensis* (Suárez-Morales et al. 2006), and *Monstrillopsis igniterra* (Suárez-Morales et al. 2008, fig. 2B). In the new species these segments are partly fused (Fig. 3A). The general armature of the antennules is similar in all the species examined, but the new species has some distinctive details: 1) the apical elements 6_1_ and 6_2_ (sensu Grygier and Ohtsuka 1995) are clearly unequally long, the former is about twice as long as element 6_2_ (Fig. 3A). In the other species these elements are equally or subequally long (Scott 1904; Sars 1921; Suárez-Morales and Ivanenko 2004; Suárez-Morales et al. 2006, 2008); 2) the new species has a unique character on the left antennule, with a supernumerary spine on the first segment, a character that has not been observed in any other monstrilloid, probably reminiscent of an ancestral armature/fusion pattern of this segment which is known to have only a single element (Grygier and Ohtsuka 1995).

*Monstrillopsis planifrons* sp. n. has a distinctive genital double-somite, with a pair of large lateral protuberances on the posterior half of the somite, visible on dorsal and ventral views (Fig. 2C, D). In its known congeners the anterior half of the genital double-somite is expanded, either strongly as in *M. igniterra* (Suárez-Morales et al. 2005, fig. 2D) and *M. chilensis* (Suárez-Morales et al. 2006, fig. 4B) or weakly as in *Monstrillopsis filogranarum* (Malaquin 1901, fig. 3), *M. ferrarii* and *M. dubioides* (Sars 1921; Suárez-Morales and Ivanenko 2004, figs. 6A, 8C). *Monstrillopsis dubia* (sensu Scott 1904) has a short, robust genital double-somite (see Scott 1904: Pl. XIV, fig. 18), expanded on its proximal half.

Another distinctive character of the new species is its produced, flat corrugate forehead; this character is absent from all its known congeners, which have a simple, rounded forehead as in *M. dubia* (Scott, 1904, pl. XII, fig. 14), *M. dubioides* (Sars 1921; Suárez-Morales and Ivanenko 2004, fig. 9E), *M. chilensis* (Suárez-Morales et al. 2006, fig. 3A, B), *M. igniterra* (Suárez-Morales et al. 2008, fig. 2D). In *M. ferrarii* the forehead is anteriorly produced and coarsely corrugate (Suárez-Morales and Ivanenko 2004, fig. 3B), thus partially resembling that of the new species; however, the anterior margin is irregular in *M. ferrarii* and completely flat in *M. planifrons* sp. n. Also, in *M. ferrarii* the cephalic area has a pair of distinctive sculptured protuberances (Suárez-Morales and Ivanenko 2004, fig. 3B, D) that are absent in the new species.

Additional differences of the new species with respect to its congeners include a weakly developed fifth leg inner lobe, which is remarkably reduced to a small rounded inner protuberance (arrowed in Fig. 3E); all the known females of the genus have a conspicuous, well-developed inner lobe, reaching beyond the mid-length of the outer lobe as in *M. ferrarii* (Suárez-Morales and Ivanenko 2004, fig. 6D,E) and *M. igniterra* (Suárez-Morales et al. 2008, fig. 2D), or slightly shorter, as in *M. dubia* (Scott 1904, pl. XIV, fig. 17), *M. chilensis* (Suárez-Morales et al. 2006) and *M. dubioides* (Suárez-Morales and Ivanenko 2004, fig. 8C). In *M. filogranarum*, described and depicted by Malaquin (1901, fig. 3), the inner lobe is present but it is weakly developed, thus resembling the pattern found in the new species. These species can be easily distinguished by differences in the length of the anal somite, which is twice as long as the preanal somite in *M. filogranarum* (Malaquin 1901, fig. 3) vs. equally long in the new species; in *M. filogranarum* the genital double-somite lacks expansions, thus diverging from the condition described in the new species.

In the new species the anal somite is about as long as the preceding urosomite; it shares this character with *M. ferrarii* (Suárez-Morales and Ivanenko 2004, fig. 6E) and *M. chilensis* (Suárez-Morales et al. 2006, fig. 2D) whereas in *M. dubia* (Scott 1904, pl. XII, fig. 14, pl. XIV, fig. 18), *M. dubioides* (Suárez-Morales and Ivanenko 2004, fig. 8E), and *M. igniterra* (Suárez-Morales et al. 2008, fig. 2D) the anal somite is longer than the preanal somite.

Finally, the new species has the cephalothorax covered by small papilla-like structures; this kind of ornamentation has not been described in other species of the genus but it is known in several species of *Monstrilla* (i.e., *M. wandelii* Stephensen, 1913; *M. elongata* Suárez-Morales, 2001; *M. pustulata* Suárez-Morales & Dias, 2001). In light of these many differences, the erection of a new species for the specimen from the Canadian Arctic seems to be well justified.

####### Habitat.

The oceanography of the lower Northwest Passage is distinctive due to its relatively lower salinity, resulting from the four large rivers draining into the waterway (Carmack and McLaughlin 2011). The salinity of the water column varied between 28.4 and 28. 8, the temperature of the water column was ca. -1.5 °C on the day of sampling. The depth of the sampling station was of 63 m and the water column was still ice and snow covered. Moreover, the region of Dease Strait has limited water exchange with its neighbouring water bodies, thus suggesting there is an accumulation of freshwater not only from rivers but also from ice melt (McLaughlin et al. 2004, Campbell et al. 2016).

## Discussion

Several authors have questioned the validity of the genus *Monstrillopsis* Sars (Davis 1949; Davis and Green 1974) whereas others have accepted it (Huys and Boxshall 1991; Boxshall and Halsey 2004; Suárez-Morales et al. 2006). The argumentation against it has relied on the presumed mixed characters shown by males of the Arctic species *M. bernardensis* from Union Strait, Bernard Harbour (Willey 1920); as in *Monstrillopsis*, these specimens have four caudal setae and the oral papilla is located anteriorly on the cephalothorax, but the specimens examined by Davis and Green (1974) from Resolute Bay, Cornwallis Island have a rudimentary fifth leg as in *Monstrilla*; they explicitly state that they differ from Willey’s population. The specimens of *M. bernardensis* from Resolute Bay should be revised and redescribed with upgraded standards since its status is confusing. For instance, the number of caudal rami should be confirmed; some species of *Monstrilla* have a small, inconspicuous caudal seta IV (i.e., *M. elongata* Suárez-Morales, 1994; *M. gracilicauda* Giesbrecht, 1893). Also, the antennulary segmentation and armature, particularly of the distal segment should be carefully examined to determine if it has the characters relatable to *Monstrillopsis*. Huys and Boxshall (1991) strengthened the genus concept by assigning to the males of *Monstrillopsis* a particular antennular type, different from those recognized in *Cymbasoma* and *Monstrilla*. With regard to the females of species of *Monstrillopsis*, little evidence contrary to the validity of the genus has been presented. The other Arctic species described by Davis and Green (1974), *M. arctica* and *M. nasuta* are both clearly species of *Monstrilla*.

This is the fourth record of a monstrilloid species and the second of *Monstrillopsis* in Arctic waters. Fontaine’s (1955) record of *M. dubia* from Ungava Bay is unconfirmed and probably pertains to a different species. It is expected that new records will arise from further examination of zooplankton samples collected in the area. Also, *M. planifrons* is described from a single specimen; all three species of *Monstrilla* found by Davis and Green (1974) from Resolute Bay were described from a few or a single specimen each.

**Figure 5.**
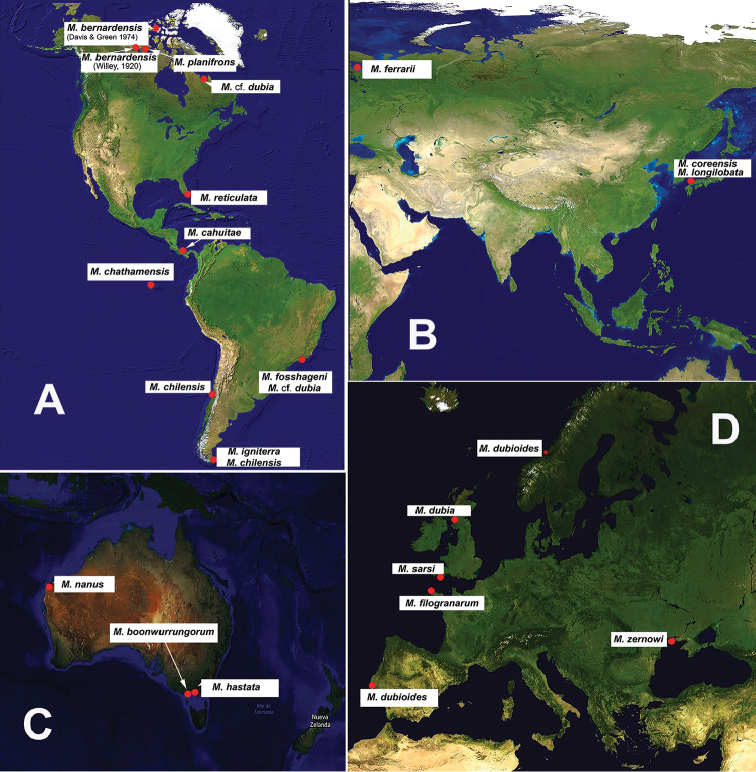
Worldwide distribution of species of *Monstrillopsis* (Scott 1904; Dolgopolskaya 1948; Davis 1949; Fontaine 1955; Suárez-Morales and Dias 2001; Suárez-Morales and Ivanenko 2004; Suárez-Morales et al. 2006, 2008; Suárez-Morales and Morales-Ramírez 2009; Suárez-Morales and McKinnon 2014; Lee et al. 2016). Only confirmed records are shown; there are no records of the genus in Africa. **A** America **B** Asia **C** Australia **D** Europe.

The most recent revision of *Monstrillopsis* (Suárez-Morales et al. 2006) resulted in the recognition of the following nominal species found at different latitudes: *Monstrillopsis filogranarum* from France (50° N), *M. dubia* from Scotland (60° N), *M. zernowi* from the Black Sea (43° N), *Monstrillopsis sarsi* from England (54° N), *Monstrillopsis fosshageni* Suárez-Morales & Dias, 2001 from Brazil (20° S), *M. dubioides* from Norway (62° N), *M. ferrarii* from the White Sea, Arctic (66° N), and *M. chilensis* from off Chile (33° S). After this revision, eight additional species of the genus were described: *M. igniterra* Suárez-Morales, Ramírez & Derisio, 2008 from the Beagle Channel (55° S), *M. chathamensis* Suárez-Morales & Morales-Ramírez, 2009, *M. cahuitae* Suárez-Morales & Carrillo, 2013 both from Costa Rica, *M. nanus* Suárez-Morales & McKinnon, 2014, *M. boonwurrungorum* Suárez-Morales & McKinnon, 2014, *M. hastata* Suárez-Morales & McKinnon, 2014 from Australia (22–38°S), *M. coreensis* Lee, Kim & Chang, 2016, and *M. longilobata* Lee, Kim & Chang, 2016 from Korea (35° N) (Suárez-Morales et al. 2008, 2013; Suárez-Morales and Morales-Ramírez 2009; Suárez-Morales and McKinnon 2014; Lee et al. 2016), most of them from male specimens. Females are known for only 7 species. The original description of *M. chilensis* Suárez-Morales, Bello-Smith & Palma, 2006 included only a female; a male found later in the same region was assigned to this species (Suárez-Morales et al. 2008). The new species described herein is therefore the 8^th^ known from a female in *Monstrillopsis*. Overall, the distribution of the genus seems to be largely restricted to temperate and cold latitudes; of the 15 known nominal species, only four (i.e., Costa Rica, Brazil, northern Australia) are known from tropical or subtropical latitudes (Razouls et al. 2005; Dias and Bonecker 2007; Suárez-Morales and Morales Ramírez 2009; Suárez-Morales and McKinnon 2014) and three (*M. ferrarii*, *M. igniterra*, *M. planifrons* sp. n.) from polar regions (Fig. 5).

Observing this adult female specimen in early June brings important new information on the distribution of monstrilloid copepods in Arctic waters as well as on their dynamics and life cycle. It also brings new information concerning the marine ecosystem of the lower Northwest Passage of the Canadian Arctic. In addition, three more observations of adult females monstrilloids were recorded during the same 2014 ICE-CAMPS campaign in Dease Strait. The specimens were collected on 07 and 29 May 2014, thus confirming that this group of copepod is consistently present in this region of the Canadian Arctic. Future work should aim to taxonomically study these three other adult females and determine if they are conspecific with *M. planifrons* to expand the knowledge of this species in the region or to determine if multiple species are present in the Dease Strait area.

## Supplementary Material

XML Treatment for Monstrillopsis planifrons
